# Date Palm Tree Leaf-Derived Cellulose Nanocrystal Incorporated Thin-Film Composite forward Osmosis Membranes for Produced Water Treatment

**DOI:** 10.3390/membranes13050513

**Published:** 2023-05-13

**Authors:** Asif Saud, Haleema Saleem, Aquib Wakeel Khan, Nazmin Munira, Maryam Khan, Syed Javaid Zaidi

**Affiliations:** Center for Advanced Material, Qatar University, Doha 2713, Qatar; asifsaud111@gmail.com (A.S.); haleemasaleem@gmail.com (H.S.);

**Keywords:** Date Palm Tree Leaf, cellulose nanocrystal, Thin-Film Composite Membrane, forward osmosis, produced water treatment

## Abstract

Worldwide water shortage and significant issues related to treatment of wastewater streams, mainly the water obtained during the recovery of oil and gas operations called produced water (PW), has enabled forward osmosis (FO) to progress and become advanced enough to effectively treat as well as retrieve water in order to be productively reused. Because of their exceptional permeability qualities, thin-film composite (TFC) membranes have gained increasing interest for use in FO separation processes. This research focused on developing a high water flux and less oil flux TFC membrane by incorporating sustainably developed cellulose nanocrystal (CNC) onto the polyamide (PA) layer of the TFC membrane. CNCs are prepared from date palm leaves and different characterization studies verified the definite formations of CNCs and the effective integration of CNCs in the PA layer. From the FO experiments, it was confirmed that that the membrane with 0.05 wt% of CNCs in the TFC membrane (TFN-5) showed better FO performance in PW treatment. Pristine TFC and TFN-5 membrane exhibited 96.2% and 99.0% of salt rejection and 90.5% and 97.45% of oil rejection. Further, TFC and TFN-5 demonstrated 0.46 and 1.61 LMHB pure water permeability and 0.41 and 1.42 LHM salt permeability, respectively. Thus, the developed membrane can help in overcoming the current challenges associated with TFC FO membranes for PW treatment processes.

## 1. Introduction

One of the most substantial forms of wastewater production related to oil and gas is produced water (PW), and it is a water-in-oil emulsion in which the surface-active agent-stabilized oil phase is properly disseminated in the aqueous phase [1]. Different materials have been developed for solving the challenging marine oil spill pollution problem [2,3]. Currently, PW is the leading wastewater type in the oil and gas sector, including an estimated worldwide volume-to-product ratio of 3:1. PW contains organic and inorganic matters, dispersed oils, suspended particles, greases, hydrocarbons, and dissolved solutes, which makes it one of the most complicated liquid wastes. The typical composition of PW includes 15 to 300 mg/L of oil and grease, 1000 to 15,000 mg/L of total dissolved solids (TDS), 20 to 2500 mg/L of chemical oxygen demand (COD), and 5 to 4000 mg/L of total suspended solids (TSS) [4]. As there is a significantly high amount of these components in PW, it can negatively impact human health and the natural environment; it is very important to properly treat the PW. Furthermore, more technical advancements are needed to comply with the stricter regulatory standards for PW discharge [5,6]. The traditional PW treatment techniques do not generally meet the discharge limits of PW, as low hydrocarbon content in the PW is not effectively separated and also specifically fails to remove oil droplets in PW that are finely scattered [7,8]. Furthermore, this traditional treatment is expensive, requires chemical use, generation of secondary waste, and only works until 60% of the water is partially recovered by using these techniques, due to its non-effective strategy in eliminating tiny oil droplets [9]. The above-stated situations have opened up the possibility of employing pressure-driven membrane approaches for the PW treatment. This membrane-based water treatment approach has been used to treat saline water effectively over the last 40 years [10,11]. Membrane technologies use a semipermeable membrane that can separate contaminants (in accordance with relative size of it to membrane pore size) whilst allowing water permeation [12].

**Figure 1:**
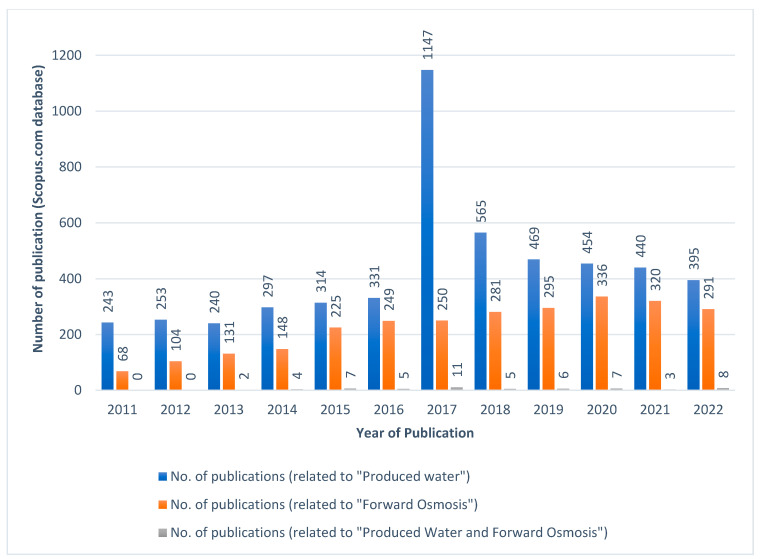
Number of publications from the Scopus database, where “Produced Water”, “Forward Osmosis”, and “Produced Water and Forward Osmosis” are seen as the keywords. Data retrieved on 14 December 2022.

Reverse osmosis (RO) is a membrane technology which relies on pressure that can treat the PW because of its capability to discriminate between oil molecules [13]. However, the fouling of the membrane, although common, makes pressure-driven membrane processes operate in a more complex way and at higher operating costs [14]. Extreme fouling is mostly caused by elevated pressure, which makes the fouling permanent. Furthermore, cleaning most membranes requires extra chemicals, energy, and treatment, which makes the pressure-driven membrane processes difficult for PW treatment [15]. Recently, FO has been utilized for the PW treatment due to the PW’s challenging nature and high fouling tendency [16]. FO is an emerging technique for separating and treating a variety of types of impure water, including PW from different sources such as oil and gas exploration and production operations [17,18]. FO can be used as a freestanding technology, or it can be combined with other methods like RO for a more effective treatment of PW [14]. FO has drawn a lot of interest as a low-fouling membrane technique due to the fact that the propensity of fouling is less serious and more reversible in FO compared to other pressure-driven membrane processes [19,20,21,22]. This is because of the “natural” transport mechanism (the osmosis phenomenon) along with the high rate of rejection of pollutants (organic and inorganic) without the application of pressure [2,17,18,19]. When compared to the dead-end filtration, the crossflow filtration allows a concentrated retention stream to run over the membrane while permeating through the pores of the membrane [23]. Hence, the FO process is associated with low energy consumption, a lower frequency of membrane fouling, simple fouling elimination, and elevated water recovery rate relative to membrane processes which require high pressure [24]. The FO technology is able to recover up to 85% of PW and operate with a feed reaching as high as 150,000 mg/L of TDS concentrations. Hence, FO processes are more reliable in PW treatment as compared to the RO process [25,26,27]. Although FO membranes exhibit less fouling than the membrane technologies utilizing pressure, their impact is nevertheless substantial [28,29]. Fouling in the FO system can be reduced through physical cleaning, chemical cleaning, back washing, air scouring, pretreatment, and antifouling coatings [30]. Pretreatment involves the use of a pre-treatment system to remove foulants before they reach the FO membrane. Common pre-treatment systems include microfiltration (MF) or ultrafiltration (UF) membranes, which can remove suspended particles and biological matter. On the other hand, FO membranes also show a low water flux, high internal concentration polarization (ICP), and external concentration polarization (ECP), and numerous research works are carried out in order to overcome these FO challenges. In the previous decades, remarkable success has been accomplished for the preparation of thin-film composite (TFC) FO membranes that consist of a polyamide (PA) salt-rejecting active layer and a mostly semi-hydrophobic support layer [31,32,33,34]. Several researchers are working on developing advanced TFC membranes for the FO process that can contribute improved water flux and reduced concentration polarization [35,36,37]. Developing appropriate FO membranes can improve overall performance of the membrane, thereby improving the overall effectiveness of the procedure. Figure 1 presents the number of publications from the Scopus database, where “Produced Water”, “Forward Osmosis”, and “Produced Water and Forward Osmosis” are seen as the keywords. It was observed that the most research related to “Produced Water” was carried out during the year 2017. Additionally, the same year witnessed the maximum sum of studies related to “Produced Water” and “Forward Osmosis”.

FO requires a lower energy consumption, which is a significant advantage in order to reduce the amount of PW as an oil/brine waste [38,39,40]. Out of the different techniques examined for PW treatment, FO was recognized as a technology that can withstand certain PW processes having increased TDS [41]. In spite of the numerous advantageous of using a TFC FO membrane for PW treatment application, it is very important to solve the related challenges such as a low water flux, a high oil flux, and the fouling tendency of the TFC membrane. Contemplating the different PW constituents, the propensity of membrane fouling will be greater compared to any other wastewater. In comparison to its CTA counterparts, the TFC membranes are more prone to fouling, especially when used with challenging feed solutions like oil/water mixtures [42,43]. This can be due to high surface roughness, strong hydrogen bonding capability, and a high initial water flux [44]. The aforementioned challenges of FO TFC membranes may be mitigated by integrating appropriate nanomaterials into the selective layer of the TFC membrane. The selective layer of the TFC membrane is the most important parameter that governs overall membrane performance [45,46]. In addition, the high rejection ability of the selective layer assures strong oil rejection, which results in high water recovery [31,34].

Cellulose nanocrystals (CNC) or nanocrystalline cellulose (NCC) are the derivatives of cellulose that are prepared through acid hydrolysis of cellulose, in which the cellulose is added to sulfuric acid under a controlled temperature as well as time period [47]. Recently, biodegradable CNC has received considerable research interest in several applications. Cellulose consisting of crystalline and amorphous areas is the critical foundation of plant fibers [48]. This can provide mechanical integrity, and therefore can be utilized to reinforce membrane materials, polymers, and bio nanocomposites [49]. CNC has a rod-like nanostructured material isolated by cellulose through the means of acid hydrolysis. CNC shows superior characteristics, such as biodegradability, biocompatibility, non-toxic nature, cost effectiveness, good mechanical properties, and high hydrophilicity [50]. These remarkable characteristics indicate the enormous possibility of CNC when integrated in water treatment membranes [51]. CNCs are currently employed in several applications, such as in water purification as adsorbents, flocculants, membranes, and absorbents [52,53]. Cellulose was incorporated in mixed matrix membranes for microfiltration and ultrafiltration; additionally, it was added in the thin-film nanocomposite (TFN) membranes in RO and FO membranes. Certain preparation techniques could increase the nanocellulose’s surface area to 500 m^2^/g. The intrinsic hydrophilic property of CNC is anticipated to increase flux and decrease the fouling propensity of the membrane. According to Rezaei-Dasht Arzhandi et al. [54], the fouling resistance and water flux of the TFC FO membrane was enhanced by the incorporation of nanocrystalline cellulose into the selective layer of the membrane. Azar et al. [32] modified PES, which was designated as the support of the TFC membrane using two different nanomaterials (CNC and NC with serine amino acid), and tested the FO performance of the membrane. It was noticed that due to the addition of CNC, the hydrophilicity of the TFN membrane amplified, which directly influenced the membrane performance with regards to water flux, salt rejection, and oil rejection. On the other hand, the modified CNC with serine amino acid again enhanced the membrane performance due to additional hydrophilic groups. In GCC countries, as date palm tree (*Phoenix dactylifera* L.) is the commonly cultivated tree, a significant volume of biowaste is produced each year during the cultivation process [55]. A proper waste extraction is necessary to convert such waste into useful products that are aimed for global reduction of environmental footprints and impacts. Date palm is abundant and rich in cellulosic fibers, and hence it can be used to prepare cellulose and other related products such as CNC [56].

In the current study, the commercial semi hydrophobic polysulphone (PSF) support layer has been used. The CNC incorporated PA layer has been synthesized via in situ interfacial polymerization (IP) process and employed in PW treatment FO process. Due to the PA active layer’s vulnerability, TFC membranes are more prone to fouling. Hence this research focused on developing an antifouling, high water flux and less oil flux TFC membrane by incorporating sustainably developed CNC to the PA layer. As nanocellulose has a high surface area, good crystallinity, surface functionalized ability, high aspect ratio, very good chemical resistance, and high Young’s modulus [53,57,58], it can help in overcoming the current challenges associated with TFC FO membranes for treating PW.

## 2. Experimental Section

### 2.1. Materials

A PSF support layer was purchased from Sigma Aldrich, St. Louis, MO, USA. The deionized water (DI), n-hexane, 1,3-phenylenediamine (MPD) (>99%), and trimesoyl chloride (TMC) (>98%) used for the IP process were bought from Merck, Kenilworth, NJ, USA. Sodium hydroxide (NaOH) extra pure pellets and sodium hypochlorite (NaOCl) and sulphuric acid (98%) for the synthesis of CNC were purchased from Sigma Aldrich. The CNC was synthesized from palm tree leaves which were obtained from the Qatar University campus. Further, in FO testing, the synthetic PW was prepared using sodium dioctyl sulfate (SDS) and diesel oil obtained from Sulphur Chemicals Pvt. Lmt Doha Qatar. Isopropyl alcohol (IPA) used for the porosity measurement was obtained from Fisher Scientific, Waltham, MA, USA.

### 2.2. Synthesis of CNC from Palm Tree Leaves

**Figure 2:**
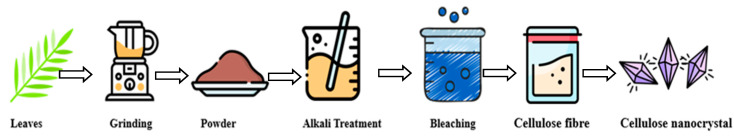
Diagrammatic representation for synthesis of cellulose microfibers.

Initially, almost 80 g of leaves were cut into little pieces (10 cm) and soaked in hot water for three hours to remove external impurities (dust, wax). The clean leaf pieces were transferred to an oven with a temperature of 90 °C for 6 h to completely evaporate the water. The dried leaves were then subjected to mechanical treatment (grinding) to transform them into powder (100–200 micrometers). The grinding procedure was used to convert the palm leaves into small fibers, which maximized the efficacy of the chemical treatment, thereby developing a favorable reaction condition by increasing the surface area of the precursor. The obtained fine powder was mixed with NaOH and water in the ratio of 1:2:10 (NaOH:Powder:Water) for alkali treatment. The solution was kept on a magnetic stirrer for 4 h at 70 °C at 1000 rpm. After 4 h of alkali treatment, it was anticipated that the palm leaf’s fibrous morphology would inflate and delignify, which was shown by a 27% weight reduction of the fibers. The resultant solution was placed undisturbed for 15 min to settle down the treated powder before being washed 10 times with 100 mL DI water. It was seen that after each wash, the dark brown color of the solution became dull. Additionally, it should be noted that the washing should not halt until the pH of the solution reaches ~7. Subsequently, the cellulose microfibrils were collected, bleached with sodium hypochlorite in a ratio of 1.5:1:10 (Sodium Hypochlorite: Powder: DI), and then placed on a magnetic stirrer at 70 °C for 3 h. The white colored microfibrous powder obtained was indeed cellulose at the end of the process. Following that, the cellulose microfibers were washed five times with 100 mL DI water, and it was noted that the whiteness became more prominent with each wash. To prevent the cellulose fibers from denaturing, the drying process should be conducted at 80 °C for 6 h. The obtained cellulose microfibers were gathered and stored in a fridge at 5 °C to avoid the moisture absorption. The cellulose microfibers were then mechanically treated (ball milling for 3 h) to reduce the size of the fibers to nano-level. The produced cellulose nanofibers (5 g) were treated with an acid by the procedure explained by Alothman and team with some modification [59]. Next, hydrolysis was performed in a solution of 40/60 (wt%/wt%) sulfuric/acetic acid containing 5 g of fiber per 100 mL of H_2_SO_4_/C_2_H_4_O_2_ solution. The hydrolysis was performed at a temperature of 55 °C for one 90 min so that the acidic reaction could react thoroughly with the fiber. The acidic hydrolyzed fiber mixture was neutralized by centrifugation (5 cycles) and the addition of distilled water to achieve a pH value of 3. The suspension was left afterwards for an hour to permit the undesirable fibers to settle. The supernatant containing CNC was then obtained by decantation. The supernatant was then freeze-dried to produce CNC powder. Figure 2 is the diagrammatic representation for synthesis of cellulose microfibers.

### 2.3. Developing the Membrane

To prepare the TFC membrane, the PSF which was used as the support was submerged initially in distilled water for at least 6 h. Subsequently, the PA layer on top of a PSF support was developed via an IP process. The membrane support was immersed in a 1 wt.% MPD/distilled water aqueous solution for 10 min, after which a synthetic rubber roller was utilized for the elimination of the surplus solution from the membrane surface. Subsequently, a 0.15 wt.% TMC–hexane solution was drained over the membrane cover for 120 s. After being exposed to air for 60 s, drying in an oven for one minute at 55 °C, and lastly being stored in deionized water. As a result, PA layer was developed on the top of the PSF support to develop a TFC membrane.

**Figure 3:**
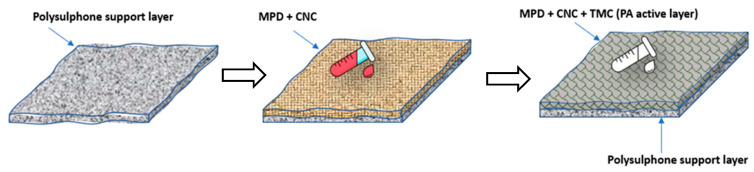
Preparation of TFN membrane.

Using the identical method as for the preparation of TFC membrane, the TFN membranes were developed (Figure 3) by mixing MPD aqueous solution with CNC at four different concentrations (0.01 wt.%, 0.03 wt.%, 0.05 wt.%, and 0.07 wt.%). The subsequent preparation process of TFM membrane stayed similar to the TFC membrane. Table 1 shows the nomenclature of the different TFN membranes developed.

### 2.4. Characterization of the Developed Cellulose Nanocrystals and Modified Membranes

CNC was characterized to verify that the nanomaterial was successfully synthesized and the characterization of both the TFC and TFN membranes was performed for examining the IP process’s impact on the membrane after nanomaterial incorporation. The FTIR (760 Nicolet) was employed for identifying the appropriate organic and inorganic peaks present in the specimen. The morphology of the CNC was examined using transmission electron microscopy (TEM) (HT 770, Hitachi, Tokyo, Japan). To understand the crystalline nature of synthesized CNC, the X-ray diffraction (Rigaku. Miniflex2 Desktop, Tokyo, Japan) was employed. The Raman Microscope Thermo Fisher Scientific DXR was the instrument used for Raman spectroscopy. The Nova Nano SEM 450 scanning electron microscope (SEM) was employed in this study for membrane characterization, and its voltage capacity ranged from 200 V–30 kV. SEM analysis was utilized to describe the surface of the membranes. For examining the surface roughness of modified and unmodified membrane specimens, a Veeco Metrology Nasoscope IV 3100 SPM has been employed. For validating the formation of the selective layer and the efficient inclusion of CNC into the TFC membranes, FTIR (760 Nicolet) analysis was carried out. The contact angle system OCA (708381-T, LMS Scientific, Selangor, Malaysia) was applied to determine the membrane’s hydrophilicity. Conductivity measurements were determined using an 856 conductivity module (Metrohm, Switzerland).

### 2.5. Preparation of Synthetic PW

**Figure 4:**
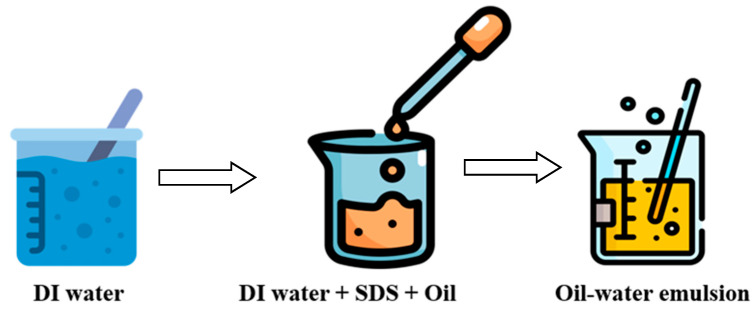
Preparation of synthetic PW.

Synthetic PW was prepared as stated by Lee and team [32] by mixing 9 parts SDS (emulsifying agent) to 1 part diesel oil to develop an oil-in-water emulsion (Figure 4), which was then homogenized with a magnetic stirrer. Finally, the mix was sonicated for 10 min in an ice bath employing a Hielscher ultrasonic processor (Hielscher UP400s, Teltow, Germany) to develop synthetic PW.

### 2.6. Membrane Porosity, Thickness

Using the gravimetric approach, the porosity of the TFC and TFN membranes was established. Initially, the membrane was submerged in 100% IPA for 1 day in order to saturate the porous structure with IPA and remove any internal water and air. The membrane was carefully switched from IPA to 50% (*v*/*v*) IPA for an additional 24 h. The membrane was weighed and dried for 1 day at ambient temperature. Then, dried membrane’s weight was calculated. The membrane porosity (ε) was determined using the Formula (1) where *ω*_1_ is the weight of the IPA wetted membrane, *ω*_2_ is the weight of dried membrane, *ρ_i_* is the density of the IPA, and *ρ_p_* is the density of the polymer. In addition, the membrane thickness was calculated using a micrometer.

(1) $$\varepsilon =\frac{\left(\omega_{1}-\omega_{2}\right)\rho_{i}}{\frac{\left(\omega_{1}-\omega_{2}\right)}{\rho_{i}}+\frac{\omega_{2}}{\rho_{p}}}$$

### 2.7. Pure Water Permeability (A), Salt Permeability (B), Salt Rejection (R_s_, %) and Oil Rejection (R_o_, %)

The water permeability (*A*, LMHB), salt rejection (*R_s_*, %), salt permeability (B, LMH), and oil rejection (*R_o_*, %) of the membranes were defined by testing the membranes employing a lab-scale dead-end filtration RO system.

The research was conducted by the experiments being at ambient temperature with a membrane active area of 0.0042 m^2^. Under a pressure of 5 bar, the *A* values have been determined by running experimentations using DI water as feed solution. The next step included collecting 5 mL of water at regular intervals and noting the duration. The experiment was done three times, and the average result was reported. The *A* value was calculated using Equation (2)

(2) $$A=\frac{V\times 60}{A_{m}\times \Delta t\times \Delta P\times 1000}$$

where, *A_m_* denotes the membrane effective area (0.00146 m^2^), *V* equals permeate volume (mL), Δ*P* is the applied pressure difference (bar), and Δ*t* is the operating duration (h), Salt rejection (*R_s_*) values were established by performing the tests using a 2000 ppm NaCl solution as the feed under a transmembrane pressure of 5 bar. An amount of 5 mL of salt permeate was collected and the time needed was documented. This process was repeated three times to obtain an average reading. *R_s_* was calculated as per Equation (3):

(3) $$R_{s}=\left(1-\frac{C_{p}}{C_{f}}\right)\times 100\%$$

where *C_p_* is the NaCl concentration of the permeate and *C_f_* is the feed NaCl concentration. These values were calculated by using the conductivity of the feed and the draw solutions.

The salt permeability (*B*) values of membranes were defined using the solution-diffusion principle as per Equation (4):

(4) $$\frac{1-R_{s}}{R_{s}}=\frac{B}{A_{m}\left(\Delta P-\Delta \pi \right)}$$

where *A_m_* is the active area of the membrane (0.00146 m^2^), Δπ is the osmotic pressure difference and ΔP is applied pressure difference.

The experiments with 20,000 ppm oil–water emulsion as the feed under a transmembrane pressure of 2 bar were used to calculate the R_o_ values. Oil rejection can be discovered in a variety of ways, such as using a UV-vis spectrometer or a TOC analyzer. The concentrations of the oil feed and permeate were obtained using a UV-vis spectrometer (DR2800, Hach) in order to obtain the oil rejection because utilizing a TOC analyzer takes more time. The absorbance was being measured at 270 nm, the wavelength at which the absorbance peaked.

*R_o_* was calculated as per the Equation (5):

(5) $$R_{o}=\left(1-\frac{C_{po}}{C_{f0}}\right)\times 100\%$$

where *C_po_* and *C_f_*_o_ represents oil permeate concentration and oil feed concentration, respectively.

### 2.8. FO Setup and Experiment (Water Flux, Reverse Salt Flux, Oil Flux)

**Figure 5:**
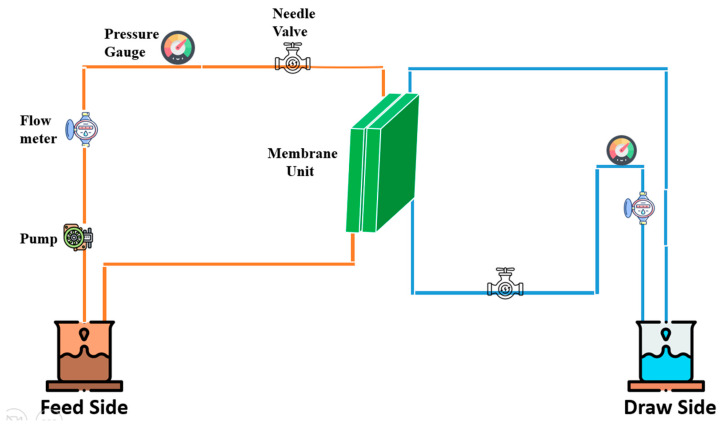
FO Experimental Setup.

As demonstrated in a prior work [7], FO experiments were performed on a crossflow forward osmosis device at lab scale. The effective membrane area in the crossflow FO cell was measured to be 0.00146 m^2^, and the system operating pressure during the process for DS and FS circulation with two variable speed pumps was observed to be 1 bar. All experiments were conducted at room temperature (22 °C). Active layer facing draw solution (AL-DS) was the membrane orientation, and 32.72 cm/s was used for the FS and DS speeds. The FS was 6000 ppm of greasy wastewater while the DS was 2.0 M of concentrated salt solution. A computerized weight balance was located at the bottom of the FS tank and was used to determine the precise water flux. An amount of 500 mL/min of cross-flow was used to circulate both FS and DS. Figure 5 depicts the configuration of the FO system used in this study.

When the system was in operation, a draw solution (2M NaCl) and a feed solution (6000 ppm) cycled counter-currently on either side of the membrane. The FO mode, in which the active layer is up against the feed solution, was used to test the membranes. Water flux (*J_v_*, LMH), reverse salt flux (*J_s_*, gMH), and oil flux (*J_o_*, gMH), which were computed using Equations (6)–(8), respectively, were used to calculate membrane performance:

(6) $$J_{v}=\frac{\Delta V}{A_{eff}\Delta t}$$

(7) $$J_{s}=\frac{\Delta \left(C_{tf}V_{tf}\right)}{A_{eff}\Delta t}$$

(8) $$J_{o}=\frac{\Delta \left(C_{td}V_{td}\right)}{A_{eff}\Delta t}$$

where *A_eff_* is the effective membrane surface area (m^2^), Δ*V* (L) is the water volume that has permeated across the membrane in a fixed time Δ*t* (h) at the time of the test. *C_tf_* and *V_tf_* are the salt concentration (g/L) and the feed volume (L) at the end of FO tests, respectively. *C_td_* and *V_td_* are the oil concentration (g/L) and the volume of the draw (L) at the end of FO tests, respectively. Using a UV-vis spectrometer (UV Biochrom Spectrophotometer) with a wavelength of 270 nm, the amount of oil in the DS was determined.

### 2.9. Forward Osmosis Experimental Results Validation

Kim and his colleagues [60] described a step-by-step process for creating a prediction model for scaling up forward osmosis in their study. When compared to the lumped parameter model prediction in the 15 °C to 35 °C temperature range, the stepwise model prediction in their work indicated a good agreement with experimental data of salt flux and water flux. The study’s findings demonstrated the advantages of using a stepwise model to forecast water flux. In the current work, certain empirical parameters in the stepwise model have been modified for specifying the osmotic pressure as well as for estimating the impact of various membrane parameters (tortuosity, porosity, salt permeability water permeability, and salt rejection).

## 3. Result and Discussion

### 3.1. Characterization of the Developed Cellulose Nanocrystals

#### 3.1.1. FTIR Analysis

**Figure 6:**
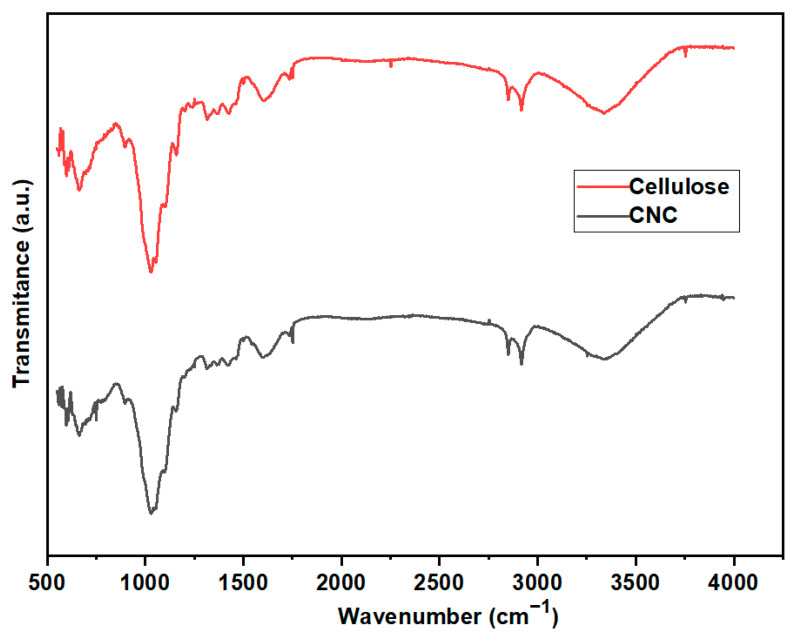
FTIR spectrum of cellulose and CNC developed.

The FTIR spectra of developed CNC and cellulose is shown in Figure 6. It should be noted that the FTIR spectra of the CNC produced and the cellulose were quite comparable. This demonstrated that no new bonds are formed during the acid hydrolysis of cellulose. The O-H bending corresponded to the spectral bands of CNC and cellulose at about 3402 cm^−1^. The C-H group, on the other hand, is responsible for the band at about 2394 cm^−1^, and the C-O bending of pure cellulose and CNC is responsible for the band at almost 1065 cm^−1^. Moreover, the cellulosic beta glycosidic linkages are associated with the bands at almost 890 cm^−1^. The C-O-C pyranose ring is responsible for the peak at almost 1050 cm^−1^, and the band at 908 cm^−1^ is connected to the glycosidic connections between the glucose units in the cellulose II framework. The cellulose and CNC FTIR results are consistent with the cellulose and CNC FTIR spectrum created by Thakur et al. [61], where the CNC was created from rice straw.

#### 3.1.2. XRD Analysis

**Figure 7:**
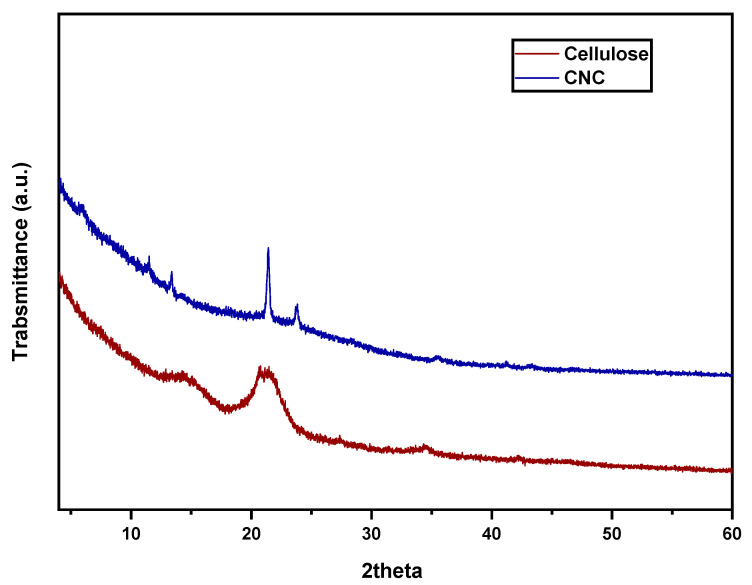
The XRD spectrum of cellulose and the developed CNC.

The crystalline behavior as well as the relationship between the properties and crystal structure are studied using the XRD analysis. Because cellulose’s molecular structure is both amorphous and crystalline, the crystalline sections of the cellulose chains are kept together by mutual hydrogen bonds, whereas the amorphous regions of the cellulosic chains do not have any hydrogen bonds. The mechanical and chemical processes have an impact on the crystallite size as well as the crystallinity of the cellulose. Figure 7 shows the CNC and cellulose XRD diffraction pattern. The samples had peaks that were consistent with the unique peaks of the cellulose framework at 2 h = 16.6 and 22.4 as well as 34.5 at the (1 1 0), (2 0 0), and (0 0 4) planes, respectively. According to the amorphous subtraction approach, the crystallinity index was found to be 61.3% for cellulose and 75.5% for CNCs. At the period of cellulose acid hydrolysis, the crystallinity index increases. The cellulose and CNC XRD results are comparable to the cellulose and CNC XRD spectra created by Thakur et al. [61].

#### 3.1.3. TEM Analysis

**Figure 8:**
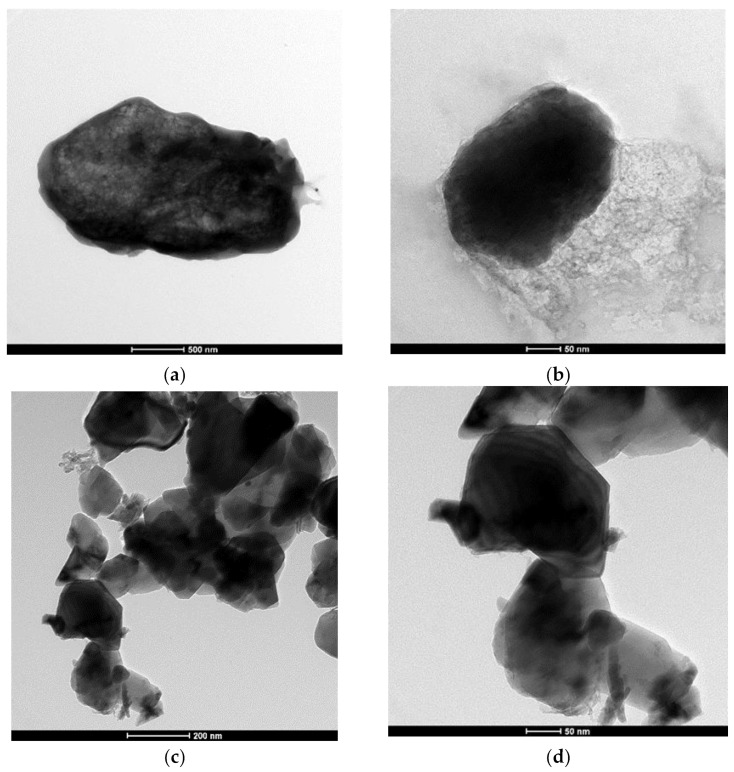
(**a**,**b**) TEM images of cellulose nanocrystals after ball milling treatment. (**c**,**d**) TEM images of acid-treated cellulose nanocrystals.

After ball milling and acid treatment, the CNC looks spherical in the TEM picture. The TEM images of cellulose nanocrystals that were treated with ball milling are shown in Figure 8a,b, respectively. The TEM images of acid-treated cellulose nanocrystals are shown in Figure 8c,d, respectively. It was shown that the CNC was only slightly aggregated, with a few particles clustering together in groups. The dimensions of cellulose nanofibers and CNC were somewhere in the 78–100 nm range. Generally, the structure of nanocellulose is determined by the conditions of acid hydrolysis as well as the source of the fiber. The structure of nanocellulose usually resembles that of a diamond crystal, but in this instance, the form appeared to be different. The ball milling effect, which breaks down the fundamental structure of nanocellulose, is the most reasonable scenario due to the changes of different shapes. The surface of the CNC formed from palm tree leaves is often coarser due to the presence of sharp edges, which might make a contribution to the formation of fouling. As compared with previous findings that were reported using the same concentration of CNC, the produced CNC do not have any jagged corners, which is still another explanation for the smooth surface combined with good nanomaterial distribution. Some examples of innovative research include the investigation of how the nanocrystal structure of cellulose is affected by a variety of mechanical processes. Researchers Agustin et al. [62], Jewan et al. [63], and Mehanny [64] found that garlic stems and palm tree leaves both produced relatively similar types of nanocellulose.

### 3.2. Membrane Characterization

#### 3.2.1. FTIR Analysis

**Figure 9:**
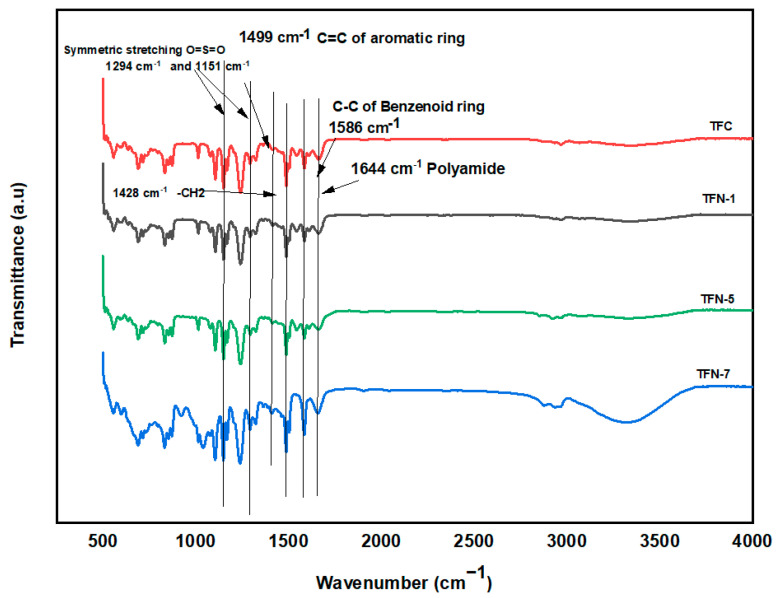
FTIR spectrum of TFC and TFN membranes.

The FTIR spectra of TFC as well as many distinct TFN membrane modifications are shown in Figure 9. At a frequency of 1645 cm^−1^, aromatic ring stretching, also known as amide II, appeared in all TFC membranes. This demonstrated that the development of the selective polyamide layer was effective. In addition, the absorption peaks that occur at roughly 1290 cm^−1^ and 1150 cm^−1^ are linked to the symmetrical bending vibrations that occur in the O=S=O group [65]. Moreover, the peaks at 1495 cm^−1^ and 1580 cm^−1^ represent distinct absorption bands of C-C stretching vibration for benzenoid rings and bands of C=C bonds for aromatic rings, respectively [59]. After incorporating CNCs in the PA layer, the spectra of all produced membranes displayed peaks at 3200 cm^−1^ and at 3500 cm^−1^ linked with the stretching of −OH groups [66]. The ATR-FITR examination of the membranes, as a result, provided conclusive evidence that CNCs are present in the TFN membranes.

#### 3.2.2. AFM Results

**Figure 10:**
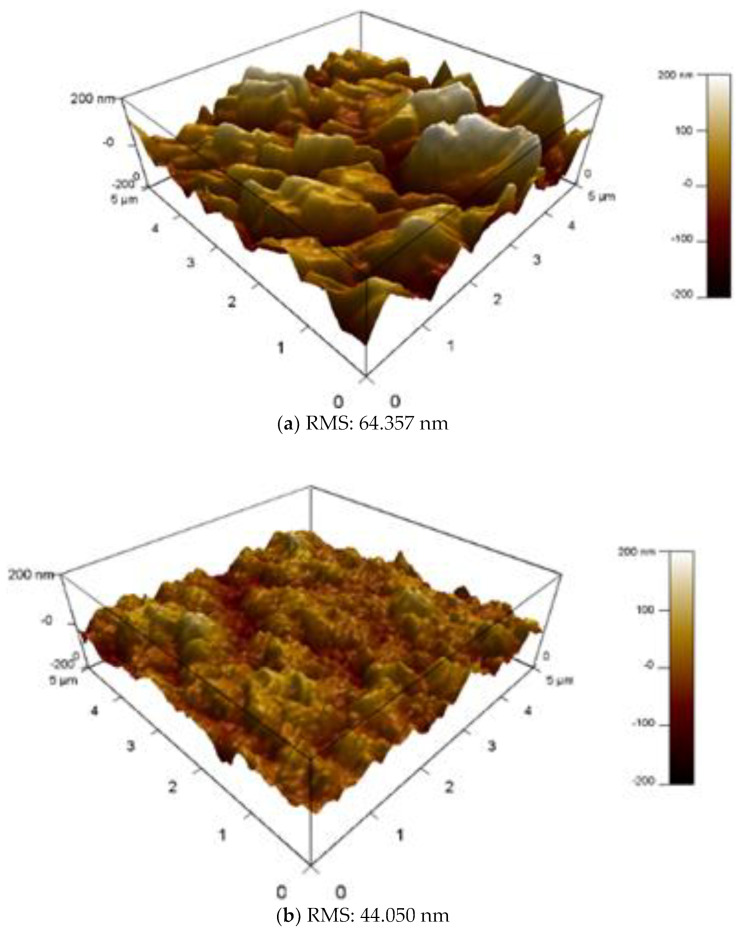

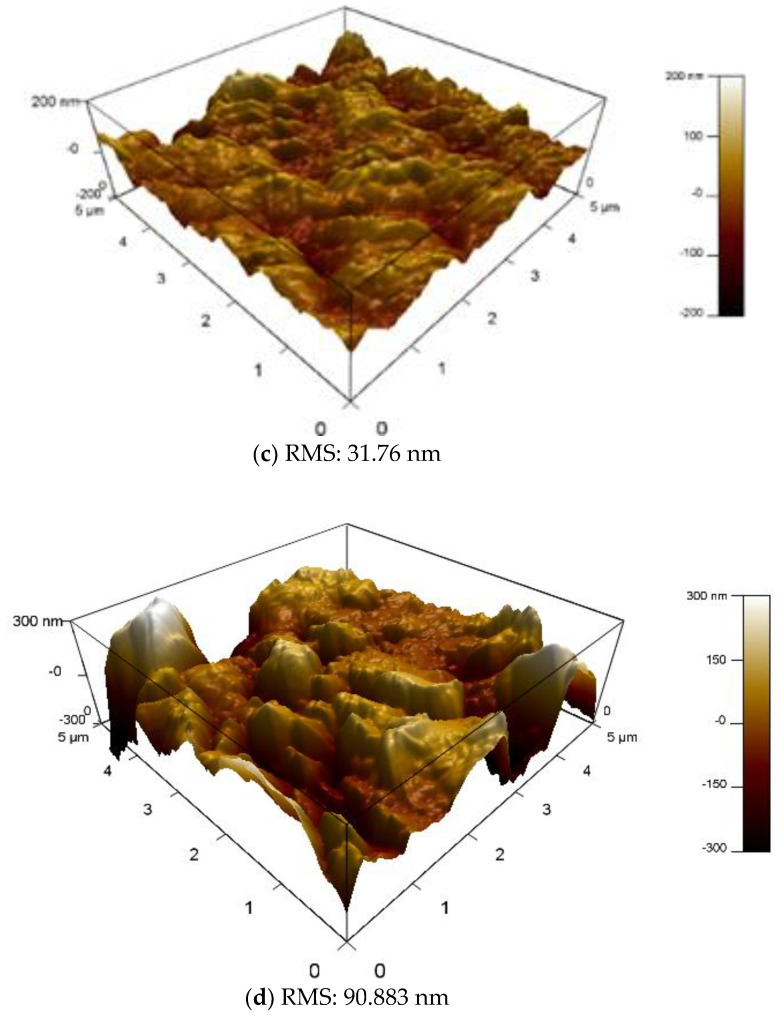
AFM images of (**a**) TFC membrane, (**b**) TFN-1 membrane, (**c**) TFN-5 membrane, and (**d**) TFN-7 membrane.

It can be observed from the findings of the AFM (Figure 10) that the surface roughness of the membranes decreased with a given CNC dosage and then increased. This indicates the morphological structure of the TFN and TFC membranes and demonstrates the amount of polymerization. This is partly related to the various concentrations of CNC that were used as well as a variation in the rate of the reaction of MPD and TMC as a result of the nanomaterial loadings [67]. The many hydroxyl groups of CNCs react with the acyl chloride group in TMC to produce ester linkages, which change the efficiency of interfacial polymerization between MPD and TMC [68]. This occurs because CNCs contain a large number of acyl chloride groups. The preparation of the polyamide layer is modified when CNCs are added, which in turn results in an alteration in the surface morphology of the TFN membranes, which have the potential to affect how well the membranes perform. The data obtained from the AFM demonstrate that, with the exception of TFN-7, all of the other unmodified and modified membranes (TFC, TFN-1, and TFN-5) maintain a thick and homogenous polyamide layer. As a result of the significant loading of CNC nanomaterials, this indicates that the degree of crosslinking was high for all of the membranes with the exception of TFN-7. These observations have an indirect and direct connection on the performance of the membrane (flux and rejections). The root mean square (RMS) roughness is one of the characteristics for membrane surface topographies that is mentioned most often in the literature. The standard deviation of the pixel height data may be thought of as the RMS roughness, which can also be thought of as the divergence of the peaks and valleys from the mean plane. The modified TFN-1,5,7 membranes each had an RMS value of 44.05 nm, 31.76 nm, and 95.88 nm, correspondingly. These figures make it very evident that the CNC has been very well integrated. Although a rougher active surface has better water permeability due to its larger surface area in contact with water, it is also more likely to become fouled because there are more possibilities for emulsified oil particles to come into contact with the large membrane surface [69]. The above makes it more likely that the membrane will become clogged. When there is a greater level of surface roughness, it is quicker for particles of a contaminant to adhere to the surface of the membrane. Therefore, membranes with a higher surface roughness experience a greater amount of fouling and a more rapid decrease in flow. As compared to the unmodified TFC membrane, the data suggest that the integration of CNC into the PA layer (TFN-1 and TFN-5) results in a reduction in the surface roughness. It was also determined that the presence of CNC nanoparticles in the proper number results in the formation of a layer that is compact, dense, and smooth, which will provide an environment that is unfavorable for the attachment of oil. The rougher the membrane, the more crests and valleys it will have. This will allow for a greater interaction with the water droplets, which will then lead to the formation of a dense and uniform hydration layer, which will increase the water flux and oil rejection because oil molecules are hydrophobic [44].

#### 3.2.3. Contact Angle Analysis

The developed membranes of contact angles are shown in the Table 2. The research demonstrated that all membranes are hydrophilic; however, the polyamide membrane coupled with cellulose nanocrystals was less hydrophobic than the other membranes. The data from the FT-IR spectrometer indicate that this is most likely due to the presence of hydroxyl -OH groups in the CNC-modified membrane. The hydrophilicity of the membranes and their roughness are shown to have a correlation with one another. The unmodified membrane with decreased roughness has a higher contact angle in contrast to the CNC-modified membrane, which exhibits a lower contact angle due to the membrane’s increased roughness. Therefore, enhanced hydrophilicity, but at the same time, the surge displayed by the TFN-7 membrane in regard to the roughness of the surface, provided evidence of irregular polymerization and agglomeration of CNCs, which led in relatively high oil and salt flow.

#### 3.2.4. SEM Analysis of Different Membranes

**Figure 11:**
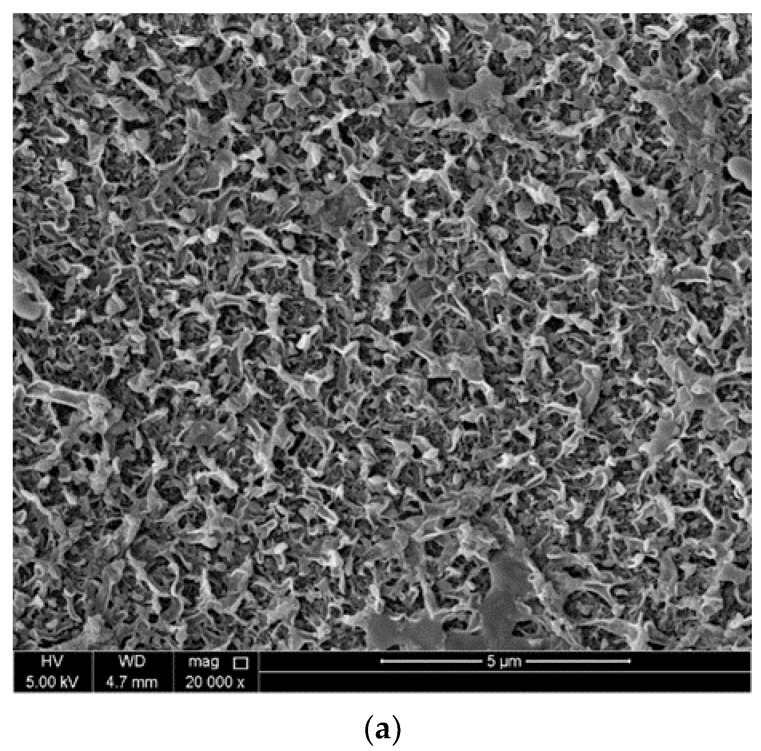

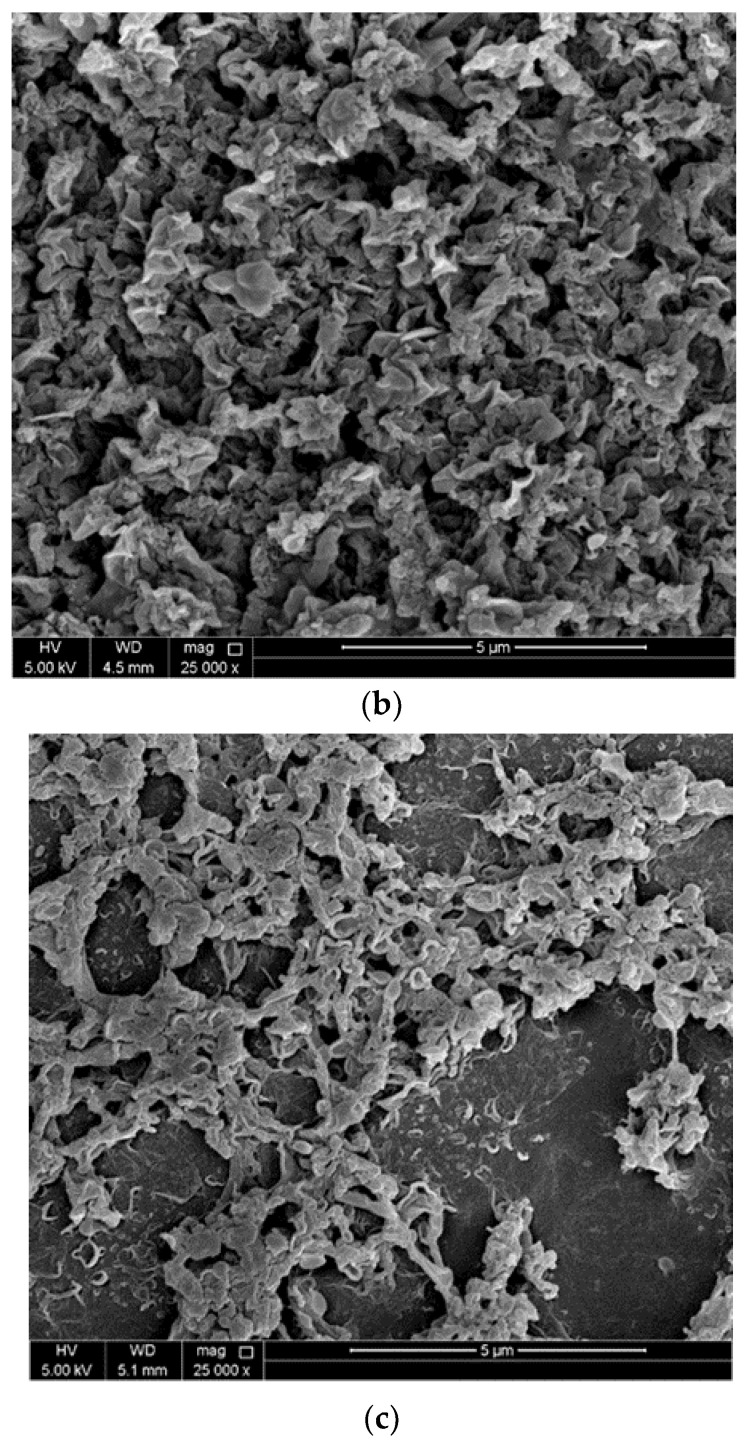
SEM images (**a**), TFC (**b**), TFN-5 membrane, and (**c**) TFN-7 membrane.

The corresponding SEM pictures of the produced membranes are shown in Figure 11. As a result of a high concentration of CNC in the PA layer, there was irregular dispersion and clustering, which resulted in a rougher PA layer deposition in membrane. This can be observed in Figure 11 and is confirmed by the data obtained from the AFM. Therefore, it is clear that raising the concentration does not instantaneously result in improved performance. Rather, the key to producing an optimum membrane that performs at its greatest potential is to strike a balance between the concentrations of the different components. On the other hand, the SEM picture obtained from TFN-5 indicated that the nanomaterials were distributed evenly across the skin layer, with certain CNC particles showing a false similarity to one another.

### 3.3. Produced Water Treatment

#### 3.3.1. Permeability and Rejections

**Figure 12:**
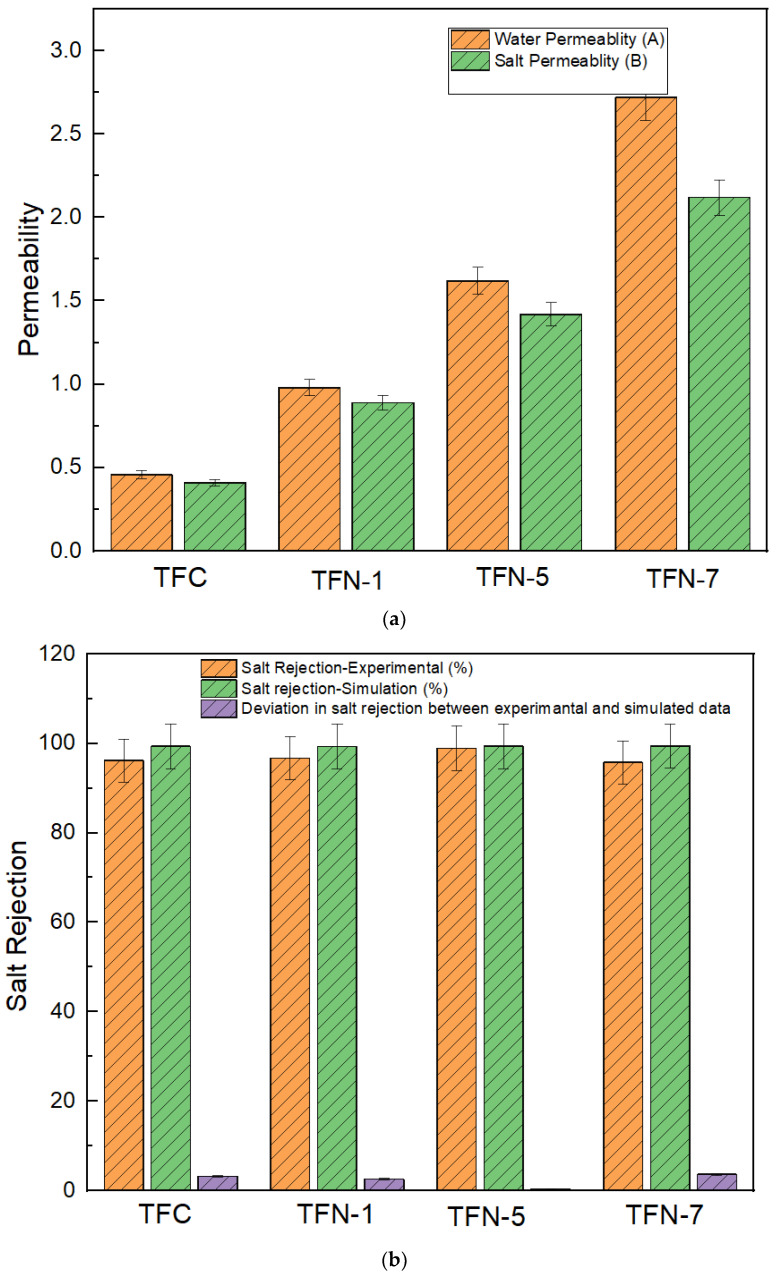

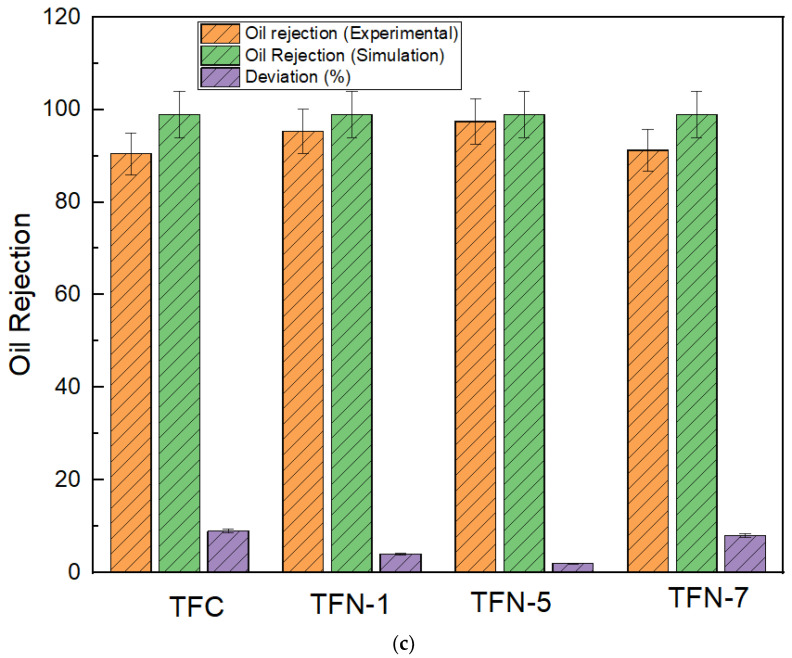
(**a**) Water permeability and salt permeability of TFC and TFN membranes. (**b**) Salt rejection (experimental), salt rejection (simulation), and percentage deviation. (**c**) Oil rejection (experimental), oil rejection (simulation), and percentage deviation.

The permeability and rejection tests were carried out using a set up that consisted of a RO dead end. The water permeability and salt permeability of TFC and TFN membranes are shown in Figure 12a. TFN-1 has 0.01 weight percent CNC in the PA layer, TFN-5 has 0.05 weight percent CNC in the PA layer, and TFN-7 has 0.07 weight percent CNC in the PA layer. When contrasted with the TFC and the other TFN membranes, it was discovered that the TFN-7 membrane has the greatest permeability coefficients for both pure water and salt. Both the water permeability and salt permeability readings were at 2.72 LMHB, whereas the salt permeability value was 2.12 LMH. On the contrary, the permeability values of the TFC, TFN-1, and TFN-5 membranes were lower than those of the TFN-7 membrane. These membranes showed values of 0.460, 1.420, and 1.61 LMHB pure water permeability (A), and 0.41, 1.24, and 1.42 LHM salt permeability (B), respectively. The phenomenon could be explained by the increased OH groups of the CNCs interacting with the increased TMC to produce an increased level of crosslinking in the selective layer of the CNC–TFC membranes. As a consequence, the membranes were able to reject more salt [70]. When the concentration of CNCs was raised to 0.07 weight percent, there was a noticeable improvement in the membrane’s flow compared to that of the uncontaminated TFC membrane. The increased flow of the membrane may be attributed to a combination of factors, including increased hydrophilicity, increased surface functional groups, and increased density of the polyamide layer. The increased surface hydrophilicity causes the membrane’s water flow to rise [71]. This is due to the stronger affinity for water, which is caused by the improved surface hydrophilicity. The presence of high-polar groups, such as -COO-, -NH_2_, and -OH on the surface of the membrane, as well as an abundance of -COO- and -OH groups on the surfaces of CNC nanoparticles, [72] contributes to an increase in surface hydrophilicity and a corresponding acceleration in the movement of water molecules. In addition, water molecules are able to travel via the interfacial gaps that exist between CNCs and the polyamide that has CNCs included in it. A passage such as this one would effectively lower the nominal diameter of the dense barrier layer of the TFC membrane, which would ultimately result in an increase in the water flow over the membrane.

Upon analyzing the levels of salt rejection (shown in Figure 12b) and oil rejection (shown in Figure 12c), it is possible to see that the TFC, TFN-1, and TFN-5 membranes all displayed greater levels of salt rejection and oil rejection, respectively. The pristine TFC membrane had a salt rejection rate of 96.2% and an oil rejection rate of 90.5%. In comparison, the TFN-1 and TFN-5 membranes had a salt rejection rate of 96.8% and 99.0%, respectively, while they had an oil rejection rate of 95.34% and 97.45%. The TFN-7 membrane, on the other hand, exhibited a lower level of rejection, which came in at 95.80% for salt and 91.22% for oil, respectively. TFN-5 showed the least deviation between simulated and experimental (salt and oil rejection data) among all TFC and TFN membranes (Figure 12b), with values of 0.4% (salt rejection) and 2.0% (oil rejection). In contrast, TFN-7 shows the highest deviation, with values of 3.68% (salt rejection) and 8.0% (oil rejection) (oil rejection). A subsequent investigation found that the heavy loading of the CNC had a detrimental effect on the growth of the thin-film PA layer, which was the primary target of the investigation. It is likely that the poor performance of TFN-7 was caused by the spontaneous, non-uniform porosity distribution throughout the surface of the membrane, as well as the uneven microporous distribution caused by the inclusion of 0.07 weight percent. Throughout the period of the interfacial polymerization process, CNC had a significant influence on the TMC and MPD polymerization occurring on the substrate. As a result, a polyamide layer with defects was produced, as seen by the scanning electron micrograph. The reduction in salt rejection was probably caused by an overloading of CNCs in the polyamide layer, which resulted in a loose structure for the polyamide material. In order to avoid the unfavorable trade-off effect that occurs during the production of the TFN-7 membrane, an adequate concentration of CNC should be used throughout this stage of the process, exactly like the formation of the TFN-1 and TFN-5 membranes.

#### 3.3.2. Water Flux, Reverse Salt Flux, and Oil Flux

**Figure 13:**
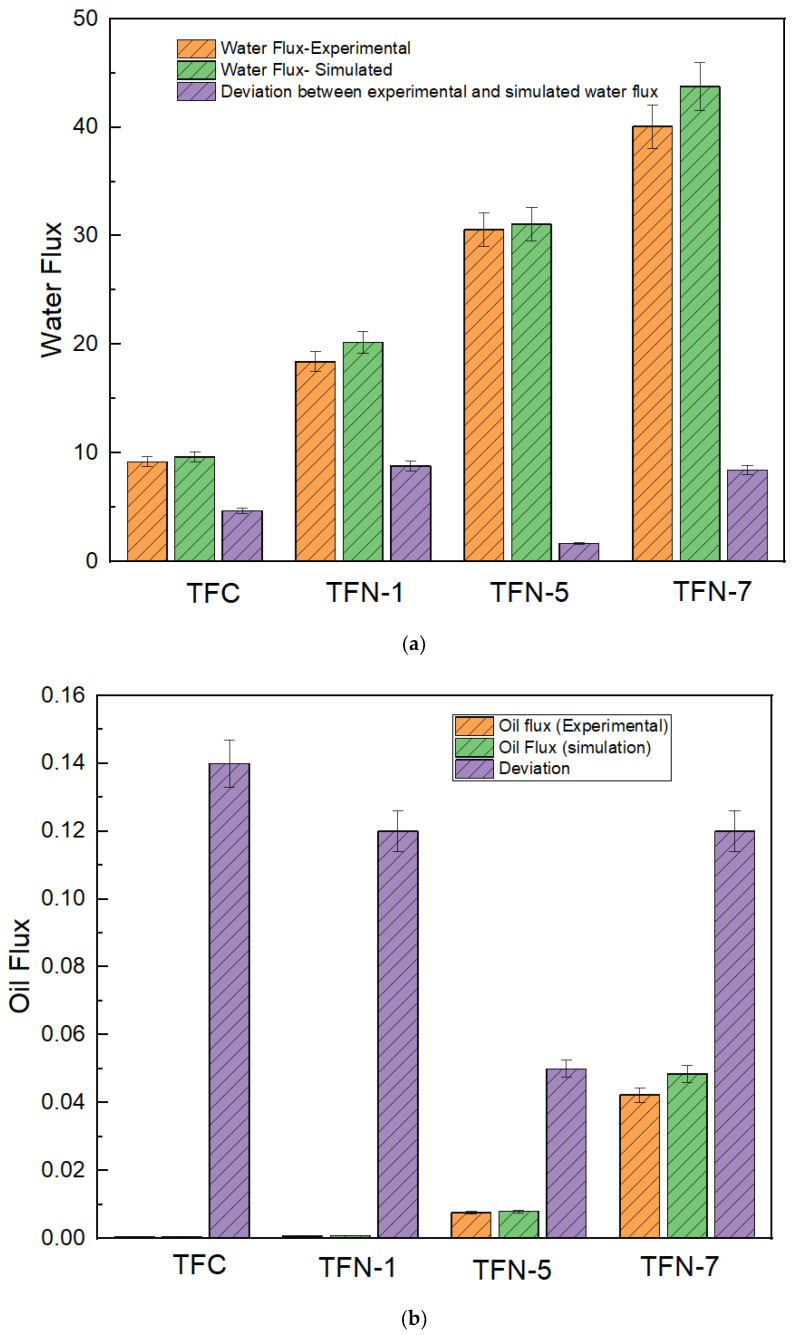

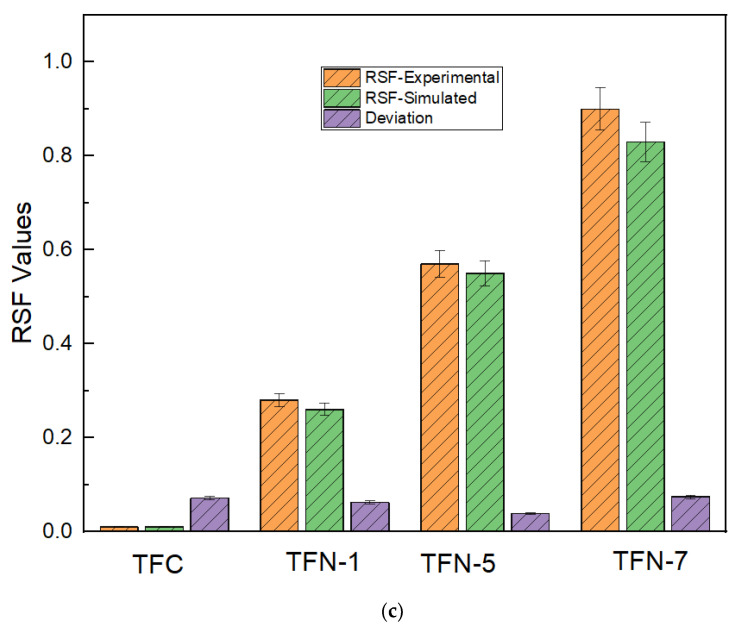
(**a**) Water flux (experimental), water flux (simulation), and percentage deviation. (**b**) Oil flux (experimental), oil flux (simulation), and percentage deviation. (**c**) RSF (experimental), RSF (simulation), and percentage deviation.

Figure 13a–c shows a comparison between the experimental, simulated, and deviation values of water, oil, and reverse solute flux of the TFC and TFN membranes, respectively. CNC is in a high concentration in the TFN-7 membrane and showed a high reverse solute flux (0.90 GMH experimental, 0.83 GMH simulated with deviation of 7.44%) and oil flux (0.00423 GMH experimental, 0.00485 GMH simulated with deviation 12.78%) as compared to TFC and other TFN membranes. This is because of a poor degree of cross-linked PA layer formation due to the high concentration of CNC, because oil rejection and salt rejection require a nicely uniform PA layer, which resists the permeation of salt and oil [72]. Due to the distorted active layer, the salt and oil flux was high. On the other hand, the TFN-5 was showing higher water flux with a higher salt and oil rejection with a lesser deviation in experimental and simulated data (30.57 LMH experimental, 30.08 LMH simulated with 1.65% of deviation). The reverse salt flux and oil flux (0.57 GMH experimental, 0.55 GMH simulated with 3.83% deviation (reverse salt flux) and 0.00754 GMH experimental, 0.00795 GMH with minimum deviation of 5.16%) are noted. The reason for high flux, salt, and oil rejection of TFN-5 was primarily the uniform dispersion of CNC with the PA layer, which allows the water molecules to interact with the inductively activated cellulose nanocrystal molecules to form a dense water layer, as water molecules [67]. The density of OH groups on the polyamide membrane is increased by the cellulose nanocrystal, increasing the membrane’s hydrophilicity. The proper dispersion of CNC into the PA layer allows a uniform pore formation condition. In addition, the internal concentration polarization of the TFN-5 membrane was also decreased, which could be due to its increased oil flux relative to other membranes. This is because of the increased deposition of solutes on pristine TFC and TFN membrane’s porous support layer, which resulted in increased internal concentration polarization, hence causing reduced water flux relative to TFN-7.

The hydrophilicity of cellulose nanocrystal plays the most important and dominant role in the performance of all modified membranes [73]. To generate flat sheets with CH-O hydrogen bonds, cellulose chains are joined together by OH-O-type hydrogen bonds. These interactions involve nonbonding forces, including electrostatic, hydrogen bonding, and van der Waals forces. Here, cellulose nanocrystals interact strongly with water because of its hydrophilic nature. Nevertheless, the intermolecular hydrogen bond losses within the chains of cellulose are replaced by new hydrogen bonds created by water molecules during the water cellulose contact. The glycosidic linkages in the cellulose molecule are surrounded by substantial anti-bonding electron clouds. The approach of the water molecules induces a polarization in these anti-bonding electron cloud structures and an electrostatic dipole can be produced by distorting electron clouds. The concept of polarizability results in the development of a wave dipole. The interactions are dominated and defined by the characteristics of the hydrocarbon chains. The Pauli Principle’s repulsion component prevents the chains from collapsing. There could be a variety of interactions, including attractive or repulsive interactions between water molecules and permanent dipoles found in cellulose polymers, induction interactions between permanent water molecule poles and induced multipoles found in cellulose chains, and any pairs of molecules resulting from interactions on instantaneous multi-poles [73]. The reason disused here is valid for all the TFN membranes but the contradiction between TFN-7 and TFN-5 membrane was generated when it comes to the salt and oil rejection efficiency, which is the main aim of the research; the lower salt and oil flux of TFN-5 membrane set a threshold point with respect to concentration of CNC.

According to the solution-diffusion theory, the FO solute flux is another essential FO parameter that is proportional to the concentration difference across the membranes. For realistic FO processes, membranes with minimal solute flux are preferred [54]. Comparing the solute flux through the CNC-nanoparticle-incorporated TFN membranes to the control TFC membrane, there was a negligible increase noted. Because of the lower PA crosslinking level, TFN-7 with the maximum CNC loading showed the largest solute flux. Since the membranes used in FO operations should have high water flux and low solute flux at the same time, it was determined that CNC loading beyond 0.05 wt% was harmful for FO applications. Since the solute flux is proportional to the salt rejection according to the solution-diffusion theory, the trend was consistent with the salt rejection reported in RO experiments [74].

#### 3.3.3. Sustainable Methods for Draw Solution Treatment

As it has been observed that the membrane is not giving 100% oil rejection, which pollutes the draw solution and again causes a major issue at a larger scale, some sustainable methods can be used to treat the draw solution, as the concentration of oil in the draw solution is very low.

1. Bioremediation: Utilizing microorganisms, such as bacteria, fungi, or algae, to break down and metabolize the oil. These microorganisms can be added to the draw solution, or one can promote the growth of indigenous microorganisms by adding nutrients like nitrogen and phosphorus. This method may take longer than other approaches but is more environmentally friendly.
2. Oleophilic materials: To remove the oil from the draw solution, oleophilic materials like oil-absorbent pads, sheets, or booms can be used. Most of these materials are hydrophobic, which means they repel water and prefer to attract oil. Once the oil has been absorbed, the oleophilic materials can be taken out and either regenerated or replaced.
3. Natural coagulants: Instead of using chemical coagulants, use more environmentally friendly ones like Moringa oleifera seeds or chitosan, which come from the shells of crustaceans. These natural coagulants can help oil droplets and solids in suspension stick together, making it easier to separate them from the draw solution.

## 4. Conclusions

In the current study, thin-film nanocomposite membranes were developed by incorporating palm-tree-leaf-derived CNC to the PA layer of the TFC membrane. CNCs were developed from date palm leaves and different characterization studies confirmed the successful formations of CNCs and the effective incorporation of CNCs in the PA layer. From the TEM analysis, the size CNC was in the range of 78–100 nm. The results from the contact angle analysis showed that all membranes are hydrophilic, but the polyamide membrane incorporated with cellulose nanocrystals was more hydrophilic. From the FO experiments, it was confirmed that that the TFN-5 membrane showed better FO performance in PW treatment. Pristine TFC and TFN-5 membrane exhibited 96.2% and 99.0% salt rejection and 90.5% and 97.45% oil rejection. Further, TFC and TFN-5 demonstrated 0.46 and 1.61 LMHB pure water permeability and 0.41 and 1.42 LHM salt permeability, respectively. The reason for high flux, salt, and oil rejection of TFN-5 was primarily the uniform dispersion of CNC with the PA layer, which allows the water molecules to interact with the inductively activated cellulose nanocrystal molecules and form a dense water layer, as the water molecules. Therefore, the fabricated TFN-5 membrane could help in overcoming the present issues related to the TFC FO membranes used in produced water treatment process.

## Figures and Tables

**Table 1:** Nomenclature and reaction conditions for the TFC/TFN membrane.

Membrane Nomenclature	MPD wt%	MPD Deposition Time (min)	TMC wt%	Curing Time (s) and Temperature °C	CNC wt (g)
TFC	1	10	0.15	60, 55	0
TFN-1	1	10	0.15	60, 55	0.01
TFN-5	1	10	0.15	60, 55	0.05
TFN-7	1	10	0.15	60, 55	0.07

**Table 2:** RMS and contact angle values of TFC and TFN membranes.

Membrane	CNC%	RMS	Contact Angle (°)
TFC	0	64.357	62.32
TFN-1	0.01	44.050	56.21
TFN-5	0.05	31.76	40.11
TFN-7	0.07	90.88	33.17

## Data Availability

Data is contained within the article.

## References

[1] Dawoud H.D., Saleem H., Alnuaimi N.A., Zaidi S.J. (2021). Characterization and treatment technologies applied for produced water in Qatar. Water.

[2] Sun H., Liu Z., Liu K., Gibril M.E., Kong F., Wang S. (2021). Lignin-based superhydrophobic melamine resin sponges and their application in oil/water separation. Ind. Crops Prod..

[3] Yuan H., Hao R., Sun H., Zeng W., Lin J., Lu S., Chen L. (2022). Engineered Janus cellulose membrane with the asymmetric-pore structure for the superhigh-water flux desalination. Carbohydr. Polym..

[4] Achilli A., Cath T.Y., Marchand E.A., Childress A.E. (2009). The Forward Osmosis Membrane Bioreactor: A Low Fouling Alternative to MBR Processes. Desalination.

[5] Lee S., Boo C., Elimelech M., Hong S. (2010). Comparison of Fouling Behavior in Forward Osmosis (FO) and Reverse Osmosis (RO). J. Memb. Sci..

[6] Mi B., Elimelech M. (2010). Organic Fouling of Forward Osmosis Membranes: Fouling Reversibility and Cleaning without Chemical Reagents. J. Memb. Sci..

[7] Alzahrani S., Mohammad A.W., Hilal N., Abdullah P., Jaafar O. (2013). Identification of Foulants, Fouling Mechanisms and Cleaning Efficiency for NF and RO Treatment of Produced Water. Sep. Purif. Technol..

[8] Coday B.D., Miller-Robbie L., Beaudry E.G., Munakata-Marr J., Cath T.Y. (2015). Life Cycle and Economic Assessments of Engineered Osmosis and Osmotic Dilution for Desalination of Haynesville Shale Pit Water. Desalination.

[9] Abounahia N.M., El-Sayed A.M.A., Saleem H., Zaidi S.J. (2023). An overview on the progress in produced water desalination by membrane-based technology. J. Water Process Eng..

[10] Duong P.H.H., Chung T.S. (2014). Application of Thin Film Composite Membranes with Forward Osmosis Technology for the Separation of Emulsified Oil-Water. J. Memb. Sci..

[11] Hickenbottom K.L., Hancock N.T., Hutchings N.R., Appleton E.W., Beaudry E.G., Xu P., Cath T.Y. (2013). Forward Osmosis Treatment of Drilling Mud and Fracturing Wastewater from Oil and Gas Operations. Desalination.

[12] Zaidi S.J., Saleem H. (2022). Reverse Osmosis Membrane Performance Degradation. J. Reverse Osmosis Syst..

[13] Putri R.E.D., Nasir S., Hadiah F. (2022). Application of Ceramic Filter and Reverse Osmosis Membrane for Produced Water Treatment. Pollution.

[14] Liu N., Yang J., Hu X., Zhao H., Chang H., Liang Y., Liang H. (2022). Fouling and chemically enhanced backwashing performance of low-pressure membranes during the treatment of shale gas produced water. Sci. Total Environ..

[15] Phuntsho S., Shon H.K., Hong S., Lee S., Vigneswaran S. (2011). A Novel Low Energy Fertilizer Driven Forward Osmosis Desalination for Direct Fertigation: Evaluating the Performance of Fertilizer Draw Solutions. J. Memb. Sci..

[16] Alamoudi T., Nawaz M.S., Obaid M., Jin Y., Soukane S., Son H.S., Gudideni V., Al-Qahtani A., Ghaffour N. (2022). Optimization of osmotic backwashing cleaning protocol for produced water fouled forward osmosis membranes. J. Membr. Sci..

[17] Higuchi H., Miyagawa M., Takaba H. (2022). Solvent—Solute Interaction Effect on Permeation Flux through Forward Osmosis Membranes Investigated by Non-Equilibrium Molecular Dynamics. Membranes.

[18] Yu Y., Zhang X., Lu P., He D., Shen L., Li Y. (2022). Enhanced Separation Performance of Polyamide Thin-Film Nanocomposite Membranes with Interlayer by Constructed Two-Dimensional Nanomaterials: A Critical Review. Membranes.

[19] Zhao Y., Duan L., Liu X., Song Y. (2022). Study on the Changes in the Microcosmic Environment in Forward Osmosis Membranes to Reduce Membrane Resistance. Membranes.

[20] Suwaileh W., Pathak N., Shon H., Hilal N. (2020). Forward osmosis membranes and processes: A comprehensive review of research trends and future outlook. Desalination.

[21] Kim Y., Li S., Ghaffour N. (2020). Evaluation of different cleaning strategies for different types of forward osmosis membrane fouling and scaling. J. Membr. Sci..

[22] Im S.J., Viet N.D., Jang A. (2021). Real-time monitoring of forward osmosis membrane fouling in wastewater reuse process performed with a deep learning model. Chemosphere.

[23] Zhao Y., Duan L., Liu X., Song Y. (2022). Influence of Membrane Fouling and Reverse Salt Flux on Membrane Impedance of Forward Osmosis Microbial Fuel Cell. Membranes.

[24] Ghazi Z.M., Rizvi S.W.F., Shahid W.M., Abdulhameed A.M., Saleem H., Zaidi S.J. (2022). An overview of water desalination systems integrated with renewable energy sources. Desalination.

[25] Salamanca M., López-Serna R., Palacio L., Hernandez A., Prádanos P., Peña M. (2022). Ecological Risk Evaluation and Removal of Emerging Pollutants in Urban Wastewater by a Hollow Fiber Forward Osmosis Membrane. Membranes.

[26] Hafiz M., Alfahel R., Hawari A.H., Hassan M.K., Altaee A. (2021). A Hybrid Nf-Fo-Ro Process for the Supply of Irrigation Water from Treated Wastewater: Simulation Study. Membranes.

[27] Wang Y., Nie Y., Chen C., Zhao H., Zhao Y., Jia Y., Li J., Li Z. (2022). Preparation and Characterization of a Thin-Film Composite Membrane Modified by MXene Nano-Sheets. Membranes.

[28] Saleem H., Goh P.S., Saud A., Khan M.A.W., Munira N., Ismail A.F., Zaidi S.J. (2022). Graphene Quantum Dot-Added Thin-Film Composite Membrane with Advanced Nanofibrous Support for Forward Osmosis. Nanomaterials.

[29] Saleem H., Saud A., Munira N., Goh P.S., Ismail A.F., Siddiqui H.R., Zaidi S.J. (2022). Improved forward osmosis performance of thin film composite membranes with graphene quantum dots derived from eucalyptus tree leaves. Nanomaterials.

[30] Altmann T., Das R. (2021). Process improvement of sea water reverse osmosis (SWRO) and subsequent decarbonization. Desalination.

[31] Hassan E., Hassan M., Abou-zeid R., Berglund L., Oksman K. (2017). Use of Bacterial Cellulose and Crosslinked Cellulose Nanofibers Membranes for Removal of Oil from Oil-in-Water Emulsions. Polymers.

[32] Lee W.J., Goh P.S., Lau W.J., Ong C.S., Ismail A.F. (2019). Antifouling Zwitterion Embedded Forward Osmosis Thin Film Composite Membrane for Highly Concentrated Oily Wastewater Treatment. Sep. Purif. Technol..

[33] Asadi A., Gholami F., Zinatizadeh A.A. (2022). Enhanced Oil Removal from a Real Polymer Production Plant by Cellulose Nanocrystals-Serine Incorporated Polyethersulfone Ultrafiltration Membrane. Environ. Sci. Pollut. Res..

[34] Han G., Chan S.S., Chung T.S. (2016). Forward Osmosis (FO) for Water Reclamation from Emulsified Oil/Water Solutions: Effects of Membrane and Emulsion Characteristics. ACS Sustain. Chem. Eng..

[35] Huang J., Ren Y., Wang X., Li H., Wang Y., Zhang J., Wang Z., Li Z., Yue T., Gao Z. (2022). Dealcoholization of kiwi wine by forward osmosis: Evaluation of membrane fouling propensity and product quality. Chem. Eng. Res. Des..

[36] Cheng X., Xu Y., Lei Z., Du J. (2022). Investigation on operational parameters and membrane fouling performance in treating synthetic aquaculture wastewater via forward osmosis with sucrose as draw solution. Sci. Total Environ..

[37] Ji C.C., Chen K.Y., Deng S.K., Wang J.X., Hu Y.X., Xu X.H., Cheng L.H. (2023). Fouling evolution of extracellular polymeric substances in forward osmosis based microalgae dewatering. Water Res..

[38] Zhan M., Gwak G., Kim D.I., Park K., Hong S. (2020). Quantitative analysis of the irreversible membrane fouling of forward osmosis during wastewater reclamation: Correlation with the modified fouling index. J. Membr. Sci..

[39] Ma C., Li Q., Liu J., Bao H., Wang L., Zhao B., Zhang Z. (2022). Forward osmosis treatment of algal-rich water: Characteristics and mechanism of membrane fouling. J. Hazard. Mater..

[40] Huang M., Liang Z., Ren L.F., Wu Q., Li J., Song J., Meng L. (2022). Robust mitigation of FO membrane fouling by coagulation-floatation process: Role of microbubbles. Desalination.

[41] Liden T., Santos I.C., Hildenbrand Z.L., Schug K.A. (2018). Treatment modalities for the reuse of produced waste from oil and gas development. Sci. Total Environ..

[42] Rahman S.N., Saleem H., Zaidi S.J. (2023). Progress in membranes for pressure retarded osmosis application. Desalination.

[43] Ding C., Zhang X., Xiong S., Shen L., Yi M., Liu B., Wang Y. (2020). Organophosphonate draw solution for produced water treatment with effectively mitigated membrane fouling via forward osmosis. J. Membr. Sci..

[44] Bell E.A., Poynor T.E., Newhart K.B., Regnery J., Coday B.D., Cath T.Y. (2017). Produced water treatment using forward osmosis membranes: Evaluation of extended-time performance and fouling. J. Membr. Sci..

[45] Yang Z., Sun P.F., Li X., Gan B., Wang L., Song X., Park H.D., Tang C.Y. (2020). A Critical Review on Thin-Film Nanocomposite Membranes with Interlayered Structure: Mechanisms, Recent Developments, and Environmental Applications. Environ. Sci. Technol..

[46] Bakly S., Ibrar I., Saleem H., Yadav S., Al-Juboori R., Naji O., Altaee A., Zaidi S.J. (2022). Polymer-based nano-enhanced forward osmosis membranes. Advancement in Polymer-Based Membranes for Water Remediation.

[47] Zhu P., Feng L., Ding Z., Bai X. (2022). Preparation of spherical cellulose nanocrystals from microcrystalline cellulose by mixed acid hydrolysis with different pretreatment routes. Int. J. Mol. Sci..

[48] Aziz T., Haq F., Farid A., Kiran M., Faisal S., Ullah A., Ullah N., Bokhari A., Mubashir M., Chuah L.F. (2023). Challenges associated with cellulose composite material: Facet engineering and prospective. Environ. Res..

[49] Siqueira G., Bras J., Dufresne A. (2010). Cellulosic bionanocomposites: A review of preparation, properties and applications. Polymers.

[50] Lalia B.S., Guillen E., Arafat H.A., Hashaikeh R. (2014). Nanocrystalline cellulose reinforced PVDF-HFP membranes for membrane distillation application. Desalination.

[51] Saud A., Saleem H., Zaidi S.J. (2022). Progress and prospects of nanocellulose-based membranes for desalination and water treatment. Membranes.

[52] Fan X.M., Yu H.Y., Wang D.C., Mao Z.H., Yao J., Tam K.C. (2019). Facile and Green Synthesis of Carboxylated Cellulose Nanocrystals as Efficient Adsorbents in Wastewater Treatments. ACS Sustain. Chem. Eng..

[53] Grishkewich N., Mohammed N., Tang J., Tam K.C. (2017). Recent Advances in the Application of Cellulose Nanocrystals. Curr. Opin. Colloid Interface Sci..

[54] Rezaei-DashtArzhandi M., Sarrafzadeh M.H., Goh P.S., Lau W.J., Ismail A.F., Wong K.C., Mohamed M.A. (2020). Enhancing the desalination performance of forward osmosis membrane through the incorporation of green nanocrystalline cellulose and halloysite dual nanofillers. J. Chem. Technol. Biotechnol..

[55] Abid W., Ammar E. (2022). Date Palm (*Phoenix dactylifera* L.) Wastes Valorization: A Circular Economy Approach. Mediterranean Fruits Bio-Wastes: Chemistry, Functionality and Technological Applications.

[56] Elseify L.A., Midani M., Shihata L.A., El-Mously H. (2019). Review on cellulosic fibers extracted from date palms (*Phoenix Dactylifera* L.) and their applications. Cellulose.

[57] Xu C., Chen W., Gao H., Xie X., Chen Y. (2020). Cellulose Nanocrystal/Silver (CNC/Ag) Thin-Film Nanocomposite Nanofiltration Membranes with Multifunctional Properties. Environ. Sci. Nano.

[58] Asempour F., Emadzadeh D., Matsuura T., Kruczek B. (2018). Synthesis and Characterization of Novel Cellulose Nanocrystals-Based Thin Film Nanocomposite Membranes for Reverse Osmosis Applications. Desalination.

[59] Alothman O.Y., Kian L.K., Saba N., Jawaid M., Khiari R. (2021). Cellulose Nanocrystal Extracted from Date Palm Fibre: Morphological, Structural and Thermal Properties. Ind. Crops Prod..

[60] Kim W.J., Campanella O., Heldman D.R. (2022). A stepwise approach to predict the performance of forward osmosis operation: Effect of temperature and flow direction. Desalination.

[61] Thakur M., Sharma A., Ahlawat V., Bhattacharya M., Goswami S. (2020). Process optimization for the production of cellulose nanocrystals from rice straw derived α-cellulose. Mater. Sci. Energy Technol..

[62] Sai Prasanna N., Mitra J. (2020). Isolation and Characterization of Cellulose Nanocrystals from Cucumis Sativus Peels. Carbohydr. Polym..

[63] Rhim J.W., Reddy J.P., Luo X. (2015). Isolation of Cellulose Nanocrystals from Onion Skin and Their Utilization for the Preparation of Agar-Based Bio-Nanocomposites Films. Cellulose.

[64] Mehanny S., Abu-El Magd E.E., Ibrahim M., Farag M., Gil-San-Millan R., Navarro J., El Habbak A.E.H., El-Kashif E. (2021). Extraction and Characterization of Nanocellulose from Three Types of Palm Residues. J. Mater. Res. Technol..

[65] Yang X., Liu H., Zhao Y., Liu L. (2016). Preparation and Characterization of Polysulfone Membrane Incorporating Cellulose Nanocrystals Extracted from Corn Husks. Fibers Polym..

[66] Sun X.F., Xu F., Sun R.C., Fowler P., Baird M.S. (2005). Characteristics of Degraded Cellulose Obtained from Steam-Exploded Wheat Straw. Carbohydr. Res..

[67] Bai L., Liu Y., Bossa N., Ding A., Ren N., Li G., Liang H., Wiesner M.R. (2018). Incorporation of Cellulose Nanocrystals (CNCs) into the Polyamide Layer of Thin-Film Composite (TFC) Nanofiltration Membranes for Enhanced Separation Performance and Antifouling Properties. Environ. Sci. Technol..

[68] Zhang R., Yu S., Shi W., Wang W., Wang X., Zhang Z., Li L., Zhang B., Bao X. (2017). A Novel Polyesteramide Thin Film Composite Nanofiltration Membrane Prepared by Interfacial Polymerization of Serinol and Trimesoyl Chloride (TMC) Catalyzed by 4-dimethylaminopyridine (DMAP). J. Memb. Sci..

[69] Mohammad A.W., Teow Y.H., Ang W.L., Chung Y.T., Oatley-Radcliffe D.L., Hilal N. (2015). Nanofiltration Membranes Review: Recent Advances and Future Prospects. Desalination.

[70] Cho K., Hill A., Caruso F., Kentish S. (2015). Chlorine resistant glutaraldehyde crosslinked polyelectrolyte multilayer membranes for desalination. Adv. Mater..

[71] Pang R., Zhang K. (2017). A facile and viable approach to fabricate polyamide membranes functionalized with graphene oxide nanosheets. RSC Adv..

[72] Isogai A., Saito T., Fukuzumi H. (2011). TEMPO-oxidized cellulose nanofibers. Nanoscale.

[73] Chami Khazraji A., Robert S. (2013). Interaction effects between cellulose and water in nanocrystalline and amorphous regions: A novel approach using molecular modeling. J. Nanomater..

[74] Ng H.Y., Tang W., Wong W.S. (2006). Performance of forward (direct) osmosis process: Membrane structure and transport phenomenon. Environ. Sci. Technol..

